# Prognostic prediction of systemic immune-inflammation status for patients with colorectal cancer: a novel pyroptosis-related model

**DOI:** 10.1186/s12957-022-02697-w

**Published:** 2022-07-14

**Authors:** Jun Hu, Caijuan Tian, Yanpeng Zhao, Yixian Guo, Shuo Chen

**Affiliations:** 1 Department of Colorectal Cancer Surgery, National Clinical Research Center for Cancer, Key Laboratory of Cancer Prevention and Therapy of Tianjin, Tianjin Medical University Cancer Institute and Hospital, Tianjin, 300060 China; 2 Tianjin Marvel Medical Laboratory, Tianjin Marvelbio Technology Co., Ltd., Tianjin, 300381 China; 3 Tianjin Yunquan Intelligent Technology Co., Ltd., Tianjin, 300381 China; 4 Department of Colorectal Surgery, The People’s Hospital of Tianjin, Tianjin, 300121 China

**Keywords:** Pyroptosis, Colorectal cancer, Tumor microenvironment, Prognosis, Therapy

## Abstract

Pyroptosis and related gasdermin family proteins play an important role in the tumorigenesis of colorectal cancer (CRC). However, the prognostic roles of pyroptosis-related genes (PRGs) and their relation to infiltrates of immune cells in the pathogenesis of CRC remain unclear. Using this study, we set up a prognostic gene pattern on the basis of 13 PRGs (AIM2, CASP1, CASP5, CASP6, CASP8, CASP9, ELANE, GPX4, GSDMD, NLRP7, NOD2, PJVK, and PRKACA) for CRC patients. A comprehensive bioinformatics analysis based on these genes was then performed. With the good AUC prediction value of the ROC curves, the group with high hazard first had a poorer survival prognosis than the group with low hazard. Second, we found that PRGs were significantly related to inflammation-associated genes and immune-associated genes in CRC. Then, we identified a correlation of PRGs with immune infiltrations in CRC. For instance, the abundances of resting NK cells resting and neutrophils were higher in the low hazard group than in the high hazard group. Overall, this work indicated that PRGs contributed to generate heterogeneity of the tumor microenvironment (TME) in CRC. This prognostic PRG model may provide a starting point for the early diagnosis and medication use of CRC.

**Supplementary Information:**

The online version contains supplementary material available at 10.1186/s12957-022-02697-w.

## Introduction

Colorectal cancer (CRC) is one of the regular causes of death worldwide. According to the appraisals of all tumor types, CRC incidence is positioned as the third cancer, and its mortality rate is positioned as the second tumor [1]. Early diagnosis and therapy of CRC can decrease CRC mortality. Mutated genes, such as PTEN, KRAS, and BRAF, are utilized as prospective biomarkers for premature diagnosis [2]. Therefore, findings of early disease biomarkers, determinations of molecular subtypes, and clarification of related genetic mechanisms may contribute to the early diagnosis and therapy of CRC, thereby improving disease prognosis.

Pyroptosis, defined as inflammatory programmed cell death, plays a significant role in the tumorigenesis of CRC [3]. Pyroptosis is a cell lytic process induced by gasdermin (GSDM). Gasdermin family proteins, which are downstream molecules of the inflammasome that are chiefly recognized for their function in pyroptosis, are also important in tumorigenesis of CRC [4]. Pyroptosis triggered by LPS can activate GSDMD to prevent progression of CRC [5]. Cell proliferation is inhibited in CRC after gasdermin C (GSDMC) downregulation, whereas cell proliferation is induced if GSDMC is upregulated. This indicates that GSDMC should be an important biomarker in the treatment of CRC [6]. Yu et al. [7] showed that gasdermin E (GSDME) interfered with the activation of caspase-3/-9 and the ROS/JNK/Bax-mitochondrial apoptosis pathway located at the downstream of lobaplatin-triggered pyroptosis.

Relations of pyroptosis to the tumor microenvironment (TME) have been established in many studies [8]. The TME is important in CRC progression. The complicated interactions between normal cells and tumor cells generate the TME. The TME can promote CRC angiogenesis and progression [9]. Immune cells infiltrating the TME can predict the prognosis of CRC [10]. A cross talk between pyroptosis and TME-related immune cells is characterized by interactions between a series of genes and cells in a synergistic manner. However, currently, many studies only focus on 1 or 2 pyroptosis-related molecules and few cell types. Therefore, a comprehensive analysis that should examine the infiltration of immune cells and several pyroptosis-related genes (PRGs) together can supply a deeper understanding of the potential mechanisms of CRC tumorigenesis.

Our work comprehensively assessed the relationship between PRG expression and the infiltration of TME-related immune cells by using 2 methods: ESTIMATE and CIBERSORT. First, 590 CRC patients were divided into 2 risk subgroups based on PRG expression profiles. Second, differentially expressed genes (DEGs) were identified between these hazard subgroups. Furthermore, we compared and analyzed mutation status, enriched gene sets, infiltrating immune cells and responses to platinum chemotherapy within these risk subgroups.

## Materials and methods

### Datasets

We used the TCGAbiolinks R package (version 2.20) to download transcriptome data of colon tumors (TCGA_COAD) and rectal tumors (TCGA_READ), in FPKM format (fragments per kilobase of exon model per million mapped fragments). Then, we transformed the FPKM type to the transcripts per million (TPM) type. Sangerbox was used to download the clinical data of colorectal cancer from TCGA, and 590 patients with colorectal cancer (CRC) containing clinical information and transcriptome data were retained.

We also downloaded somatic mutation data of colorectal cancer from the central webpage of TCGA (https://portal.gdc.cancer.gov/). Based on merged information of the transcriptome dataset, clinical dataset, and mutation dataset, the data of 526 colorectal cancer patients including clinical data, transcriptome data, and mutation data were retained. Data including 590 cases were used only for establishing the prognostic risk model, and data including 526 cases were used in other subsequent analyses.

### Establishment of a prognostic risk model for CRC

The transcriptome data were classified by chance into a modeling group and a test group based on the ratio of 7:3. In the modeling group, Cox modeling was constructed by pyroptosis-related genes (PRGs) of CRC, and stepwise regression was used to screen variables. According to the calculations of the Cox modeling as well as the mean risk score, patients were classified into two groups: the high hazard score group and the low hazard score group. The survival R package (version 3.2–3) and survminer R package (version 0.4.8) were used for Cox univariate and multivariate analyses of the modeling group and test group, and survival curves were drawn. The ROC curves of the modeling group and test group data were produced by using the PROC R package (version no. 1.17.0.1) in R software (Windows version 3.6.3). The area below the ROC curve evaluates the authenticity and reliability of Cox modeling. The larger the area under the curve is, the higher the resolution.

### Mutation data analysis

Mutation data were shown by using the “Maftools” R package, and then related oncoplots were produced. Based on expression data and mutation data of PRGs of CRC, the relationship between mutation of PRGs and PRG expression was tested. The relationship with *p* less than 0.05 was selected to draw a statistical boxplot. According to the prognosis risk model for CRC, oncoplots of mutation data were split into a high hazard score group and a low hazard score group.

### Gene set enrichment analysis (GSEA)

GSEA was applied to CRC data to determine biological pathways that were considerably modified between the high hazard score group and the low hazard score group. GSEA with Java format (version 4.0.3) was used, and sets of genes “h.all.v7.2.symbol.gmt” and “c5.all.v7.2.symbol.gmt” were selected as the reference. The following threshold (NOM *p*-value < 0.05, |NES| > 1, FDR *q*-value < 0.25) was adopted to statistically enrich biological pathways.

### Differentially expressed genes (DEGs)

The R package “Limma” was applied to conduct differential expression analysis between groups with 2 levels of hazard score. DEGs were identified based on the cutoff threshold adj. *p*-value < 0.05 as well as (|logFC| > 1). GO enrichment analysis was followed to be performed.

### Association analysis between the PRGs and inflammation-related genes in CRC

Gene set variation analysis (GSVA) was first applied to analyze Spearman correlation between 100 inflammation-related genes and 35 PRGs. Then, the gene expression profile was used to analyze the Spearman correlation between 100 inflammation-related genes and 35 PRGs, and a heatmap was drawn. To further characterize interaction relationships, highly correlated pairs (correlation coefficients were more than 0.65) were selected to set up protein-protein interaction (PPI) networks by applying the STRING database (https://string-db.org/).

### Immune feature analyses of the PRGs signature in CRC

CIBERSORT analysis was performed based on gene expression data, file “LM22.txt” (downloaded from “Supplementary Table 1” of CIBERSORT paper: https://www.nature.com/articles/nmeth.3337#MOESM207), and R code of Cibersort (https://rdrr.io/github/singha53/amritr/src/R/supportFunc_cibersort.R). Boxplots were generated to describe different components of immune cells between groups with 2 levels of hazard score. On the basis of the estimate R package (1.0.13), estimate analysis was performed. According to gene sets of immune cells, GSVA followed by drawing boxplots was performed. Finally, a heatmap describing the Spearman correlation between 100 immune-related genes and 35 PRGs was drawn.

### Pyroptosis risk score analysis in the responses to platinum chemotherapy in CRC

TCGA clinical data were screened to identify patients with/without platinum-based chemotherapy. Kaplan-Meier survival curves were drawn to analyze survival rates between groups with 2 hazard scores in the patients with/without platinum-based chemotherapy.

### Statistical analysis of statistics

We adopted R software version 4.1.0 to conduct statistical analysis. Normally, distributed data were analyzed by applying an unpaired *t*-test. Nonnormally, distributed data were analyzed by applying the Wilcoxon rank-sum test. The analysis of counting data was conducted by applying the chi-square test or Fisher’s exact probability method.

## Result

### Establishment of Cox regression model for colorectal cancer (CRC)

**Fig. 1:**
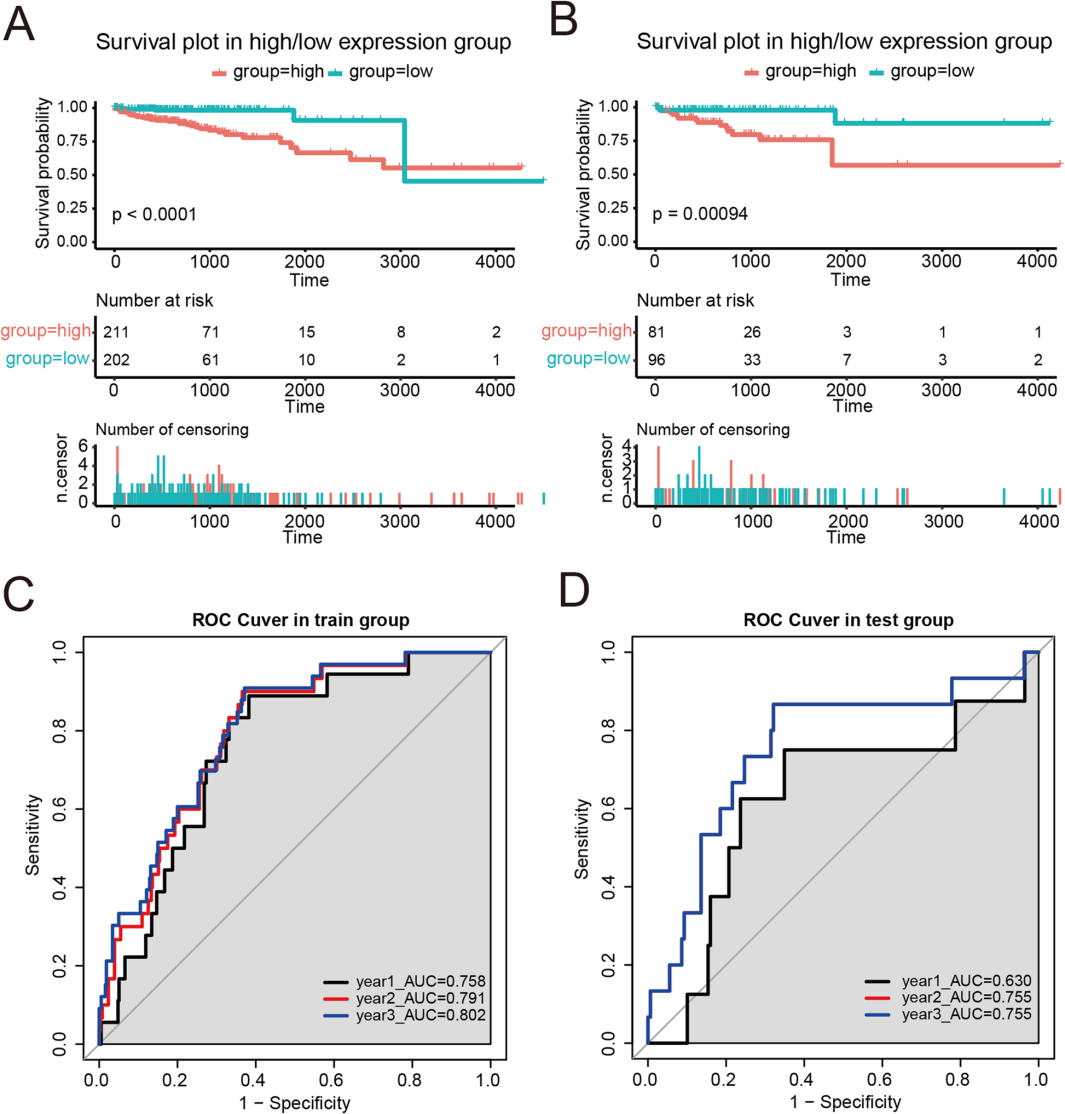
Establishment of a Cox regression model for CRC. **A** and **B** Overall survival plots for CRC patients in the high-/low-hazard group. **C** and **D** ROC curve for determining the forecast value. PRG, pyroptosis-related gene; CRC, colorectal cancer

Using the Cox regression model, we predicted the risk of colorectal cancer (CRC) including colon tumors (TCGA_COAD) and rectal tumors (TCGA_READ). Transcriptome data from COAD and READ datasets were randomly classified into a training group (70% samples, total 413 cases) and a test group (30% samples, total 177 cases). By using multivariate linear regression analyses and stepwise regression analysis, 13 pyroptosis-related genes (PRGs) were selected, and harzard modeling was generated on the basis of these genes. The formula of the modeling for colon and rectal cancer is risk_score = 0.116011 + 0.004995 × AIM2-0.000642 × CASP1 + 0.002337 × CASP5 + 0.000557 × CASP6-0.005383 × CASP8-0.007034 × CASP9-0.040302 × ELANE + 0.000154 × GPX4-0.000887 × GSDMD-0.116512 × NLRP7-0.004866 × NOD2 + 0.050655 × PJVK + 0.002196 × PRKACA. We obtained 2 groups (high-hazard group and low-hazard group) by using the median hazard score. In the training and test sets, the group with a high hazard score had a poorer survival prognosis than the group with a low hazard score (Fig. 1A and B). The AUC prediction values of the ROC curves at 1 year, 2 years, and 3 years in the training set were 0.758, 0.791, and 0.802, respectively, whereas the AUC prediction values of the ROC curves at 1 year, 2 years, and 3 years in the test set were 0.630, 0.755, and 0.755, respectively. All the above data demonstrated that this harzard model based on pyroptosis-related genes has great predictive capacity for the prognosis of CRC patients.

### Mutant profiles of PRGs in CRC

**Fig. 2:**
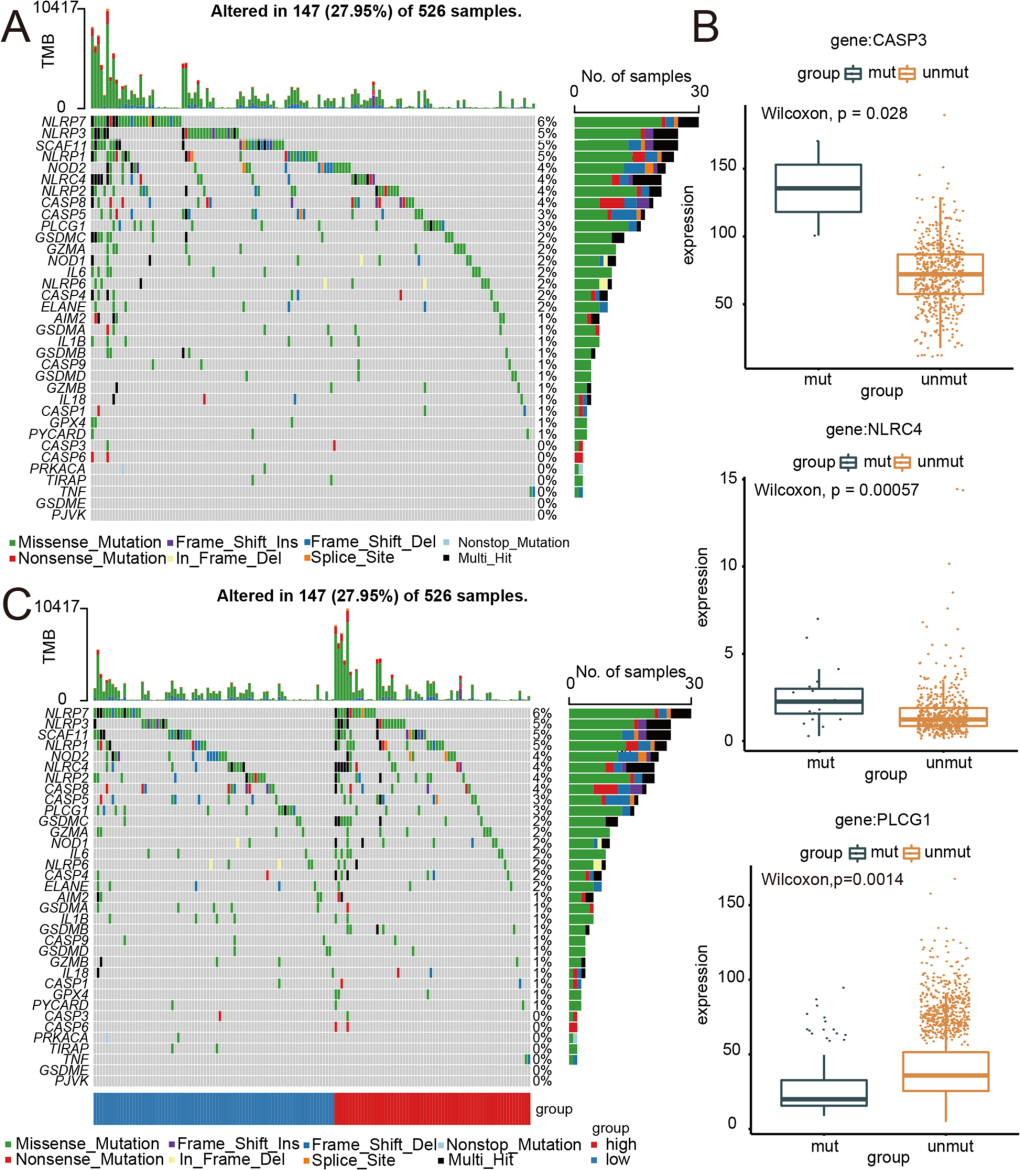
Landscape of mutation information of PRG in CRC. **A** The mutation frequency and categorization of 35 PRGs in CRC. **B** Boxplot of PRG expression versus PRG mutation status. **C** The mutation frequency and categorization of 35 PRGs in the high-/low-risk group

We overviewed the whole picture of somatic mutants as well as copy number mutants of 35 PRGs in CRC. As demonstrated in Fig. 2A, 147 of 526 (27.95%) CRC samples showed genetic mutations. Missense mutation was the most frequent mutant category (Fig. 2A). The results showed that NLRP7 had the highest mutation frequency, followed by NLRP3 and SCAF11, among the 35 PRGs (Fig. 2A). Furthermore, the Wilcoxon rank-sum test was employed to evaluate the effects of genetic mutations of PRGs on the expression profile of PRGs in CRC. When compared to mutant samples, we found that the expression levels in 3 of 35 PRGs of unmutated samples were significantly differentially regulated (Fig. 2B, CASP3, NLRC4, PLCG1). Finally, we compared genetic mutations of 35 PRGs between groups with 2 levels of hazard score. There was a difference between these 2 groups in the mutant category for 35 PRGs (Fig. 2C).

### Pathways involved in the Cox regression model for CRC

**Fig. 3:**
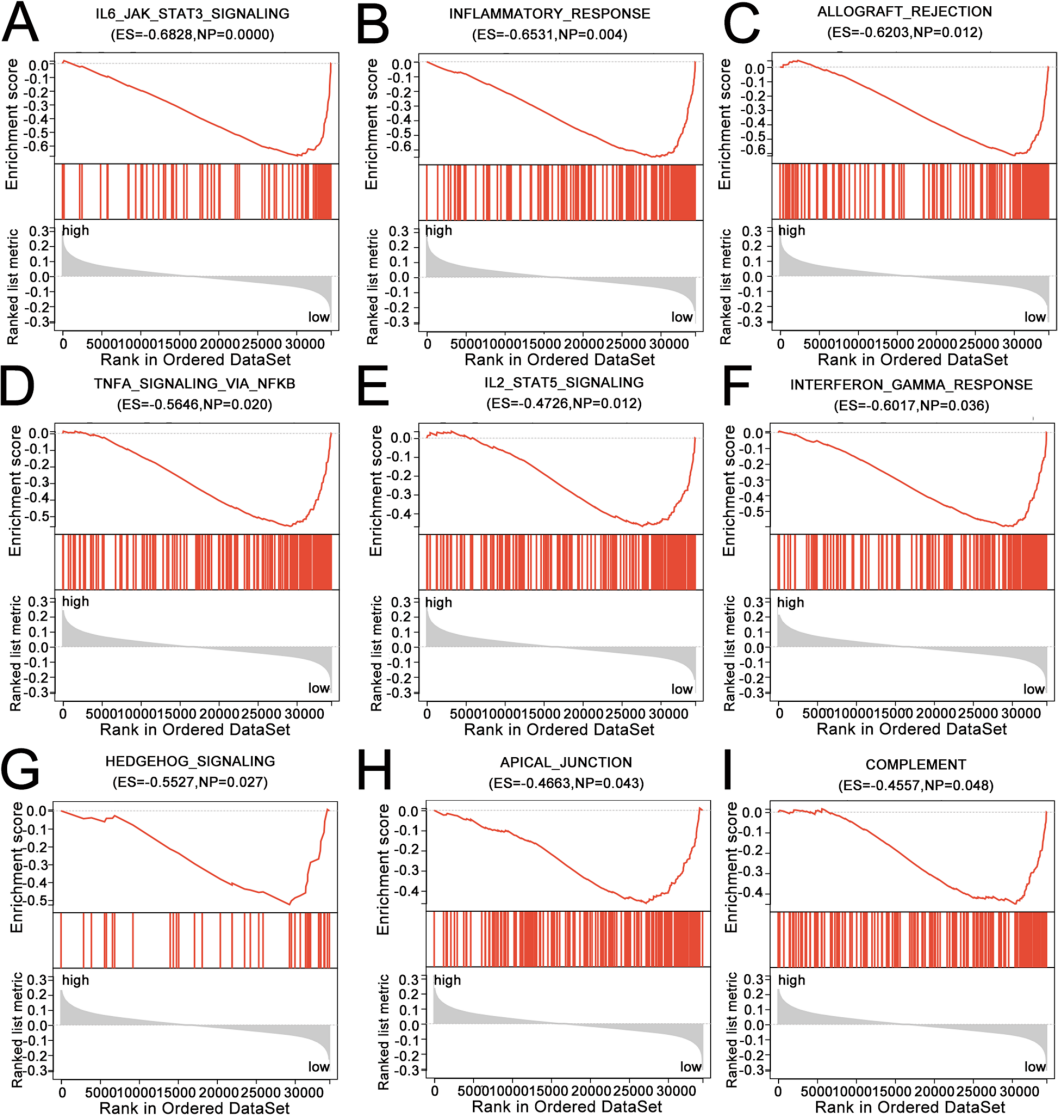
GSEA explores pathways involved in the Cox regression model for CRC. **A**–**I** Low risk scores were significantly related to “IL6_JAK_STAT3_SIGNALING,” “INFLAMMATORY_RESPONSE,” “ALLOGRAFT_REJECTION,” “TNFA_SIGNALING_VIA_NFKB,” “IL2_STAT5_SIGNALING,” “INTERFERON_GAMMA_RESPONSE,” “HEDGEHOG_SIGNALING,” “APICAL_JUNCTION,” and “COMPLEMENT”

GSEA was employed to explore the pathways involved in Cox regression modeling of CRC. In Fig. 3A–I, low-risk scores were significantly related to “IL6_JAK_STAT3_SIGNALING,” “INFLAMMATORY_RESPONSE,” “ALLOGRAFT_REJECTION,” “TNFA_SIGNALING_VIA_NFKB,” “IL2_STAT5_SIGNALING,” “INTERFERON_GAMMA_RESPONSE,” “HEDGEHOG_SIGNALING,” “APICAL_JUNCTION,” and “COMPLEMENT.”

### Determination of differentially expressed genes (DEGs) within pyroptosis subgroups

**Fig. 4:**
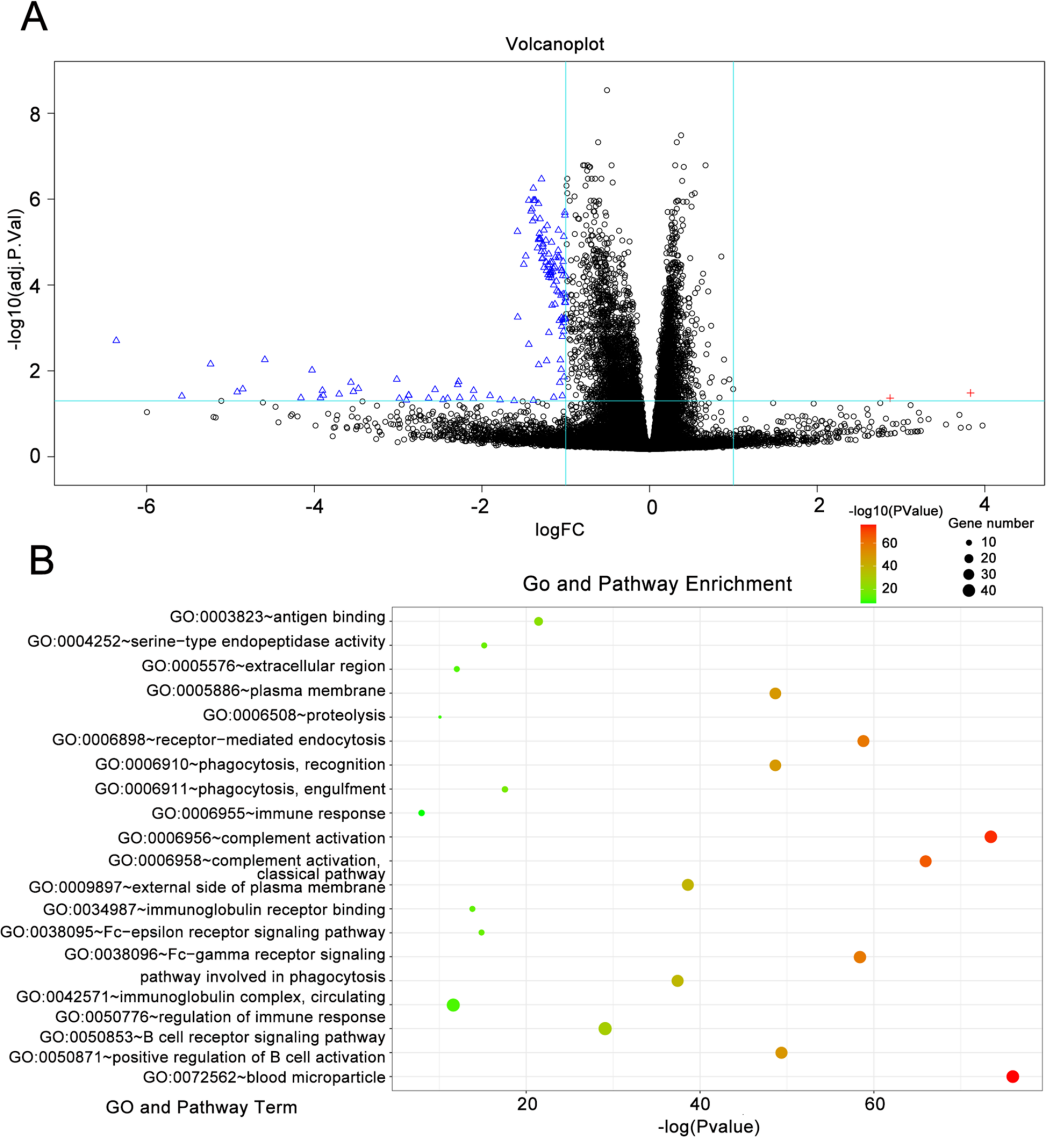
Differentially expressed genes (DEGs) in the high-/low-hazard group. Volcano plot (**A**) showing differentially expressed genes (DEGs) between groups with 2 hazard scores. Red symbols denote upregulated DEGs, blue symbols denote downregulated DEGs, and black symbols denote nondifferentially expressed genes. **B** Gene functional enrichment of dysregulated expressed genes

To clarify the gene expression pattern of pyroptosis subgroups, we used the R package “Limma” to conduct analysis of differential expression between groups with 2 levels of hazard score. Based on the cutoff threshold (adj. *p*-value < 0.05 as well as |logFC| > 1), we identified the complete all 133 DEGs, including 2 upregulated and 131 downregulated DEGs (Fig. 4A). The differentially expressed DEGs were mostly significantly associated with “antigen binding,” “complement activation, classical pathway,” and “complement activation” among the GO and pathway terms (Fig. 4B).

### Association between pyroptosis-associated genes and inflammation-associated genes in CRC

**Fig. 5:**
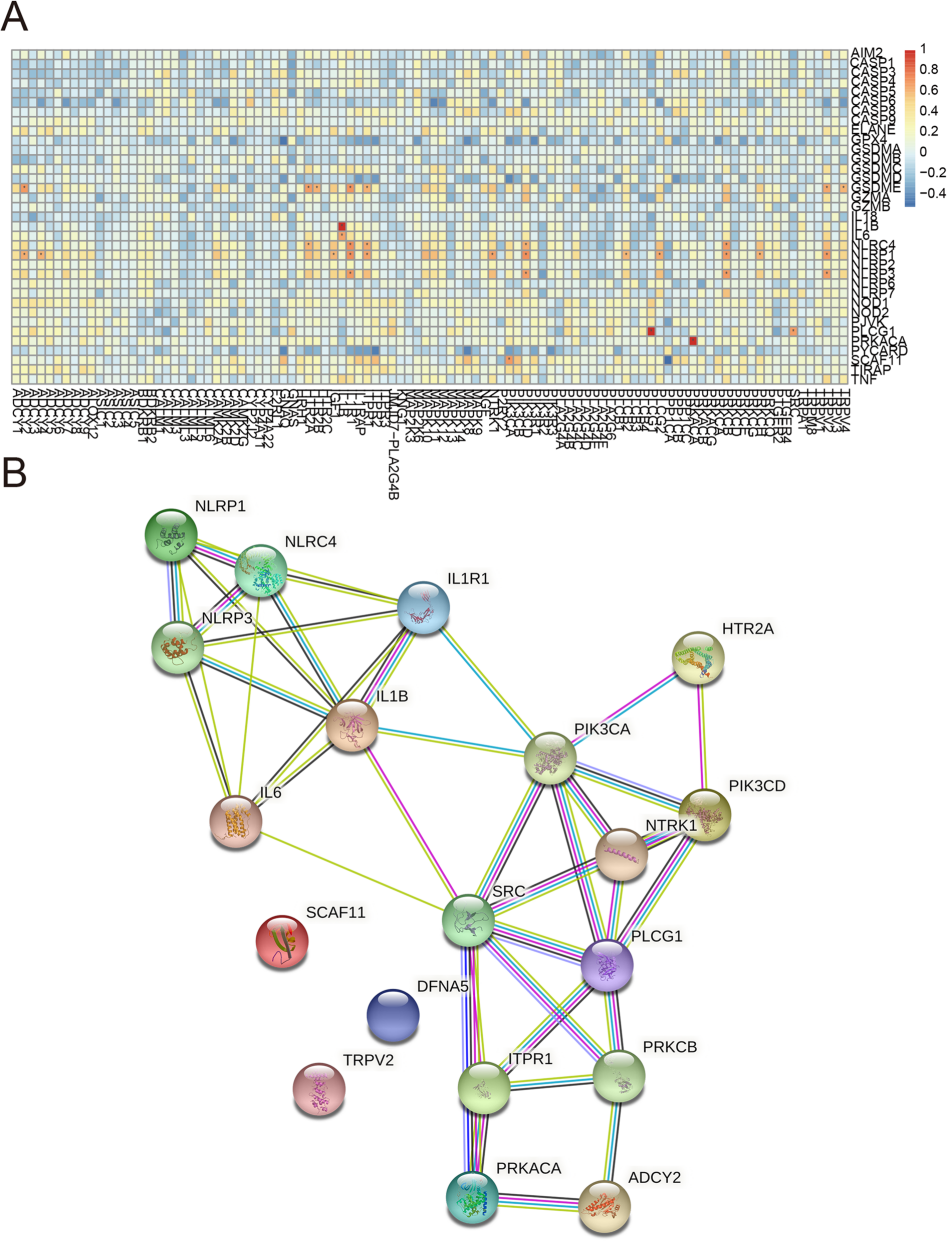
Association between the PRGs and inflammation-related genes in CRC. **A** Spearman correlation analysis between 100 inflammation-related genes and 35 PRGs. **B** Protein-protein interaction (PPI) network of inflammation-related genes and associated PRGs

We then evaluated whether pyroptosis-associated genes are correlated with inflammation-associated genes in CRC. We selected gene set variation analysis (GSVA) to caculate the Spearman correlation between 100 inflammation-related genes and 35 pyroptosis-related genes. To our surprise, no relevance was found between these 2 gene sets. However, when we used the gene expression profile to analyze the Spearman correlation between 100 inflammation-related genes and 35 pyroptosis-related genes, we found certain relationships existed. For instance, PRGs (GSDME, NLRC4, NLRP1, etc.) were shown to be significantly associated with several inflammation-related genes in the heatmap (Fig. 5A). To further characterize interaction relationships, we selected highly correlated pairs (correlation coefficients were more than 0.65) to set up protein-protein interaction (PPI) networks by applying the STRING database (https://string-db.org/). Both positive activation interactions and negative inhibition interactions revealed relationships between these inflammation-related genes and pyroptosis-related genes (Fig. 5B).

### Immune feature evaluations of the signature of pyroptosis-related genes in CRC

**Fig. 6:**
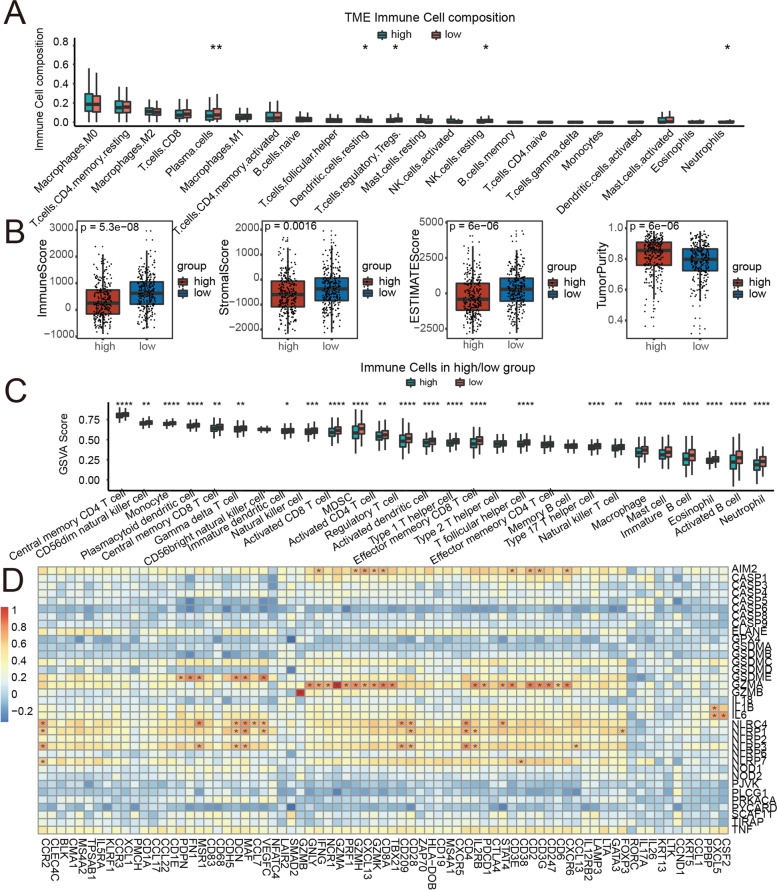
Association between the PRGs and immune features in CRC. **A** Relationships between the high-/low-hazard scores and infiltration abundances of 22 types of immune cells. Relationships between the high-/low-hazard scores and immune score (**B**), stromal score (**C**), ESTIMATE score (**D**), and tumor purity (**E**). **F** The ssGSEA of infiltrating modes of immune cells in the high- and low-hazard score groups. **G** Spearman correlation analysis between 100 immune-related genes and 35 PRGs

Then, we assessed the infiltrate status of immune cells and found that plasma cells, regulatory T cells, resting NK cells, and neutrophils were present in the low-hazard group, whereas the high-hazard group was enriched with resting dendritic cells (Fig. 6A). In the current results, the ESTIMATE score also showed that the immune score of the high-hazard group was lower than that of the low-hazard group (Fig. 6B). In addition, stromal score and ESTIMATE score were considerably lower in the high-hazard group than in the low-hazard group, while the tumor purity was conversely higher (Fig. 6C, D, E). We next used the ssGSEA algorithm to detect infiltrating modes of immune cells within two divergent sections. Except for type-2 T-helper cells, effector memory CD4 T cells, CD56 toxin natural killer cells, and memory B cells, we found that the patients in the low-hazard group had considerably higher levels of immune cell infiltration by the other 24 kinds of immune cells than the patients in the high-hazard group (Fig. 6F). Finally, we used the gene expression profile to analyze the Spearman correlation between 100 immune-related genes and 35 pyroptosis-related genes. We found certain relationships, in which we labeled highly correlated pairs (correlation coefficients were more than 0.6) with stars. For instance, PRGs (GSDME, NLRC4, GZMA, etc.) were shown to be significantly associated with several immune-related genes in the heatmap (Fig. 6G). Our study showed that pyroptosis risk scores were associated with immune features, and that increased immune reaction in the low-hazard group may be involved in the antitumor immunity of CRC.

### Pyroptosis risk score in the function of platinum chemotherapy in CRC

**Fig. 7:**
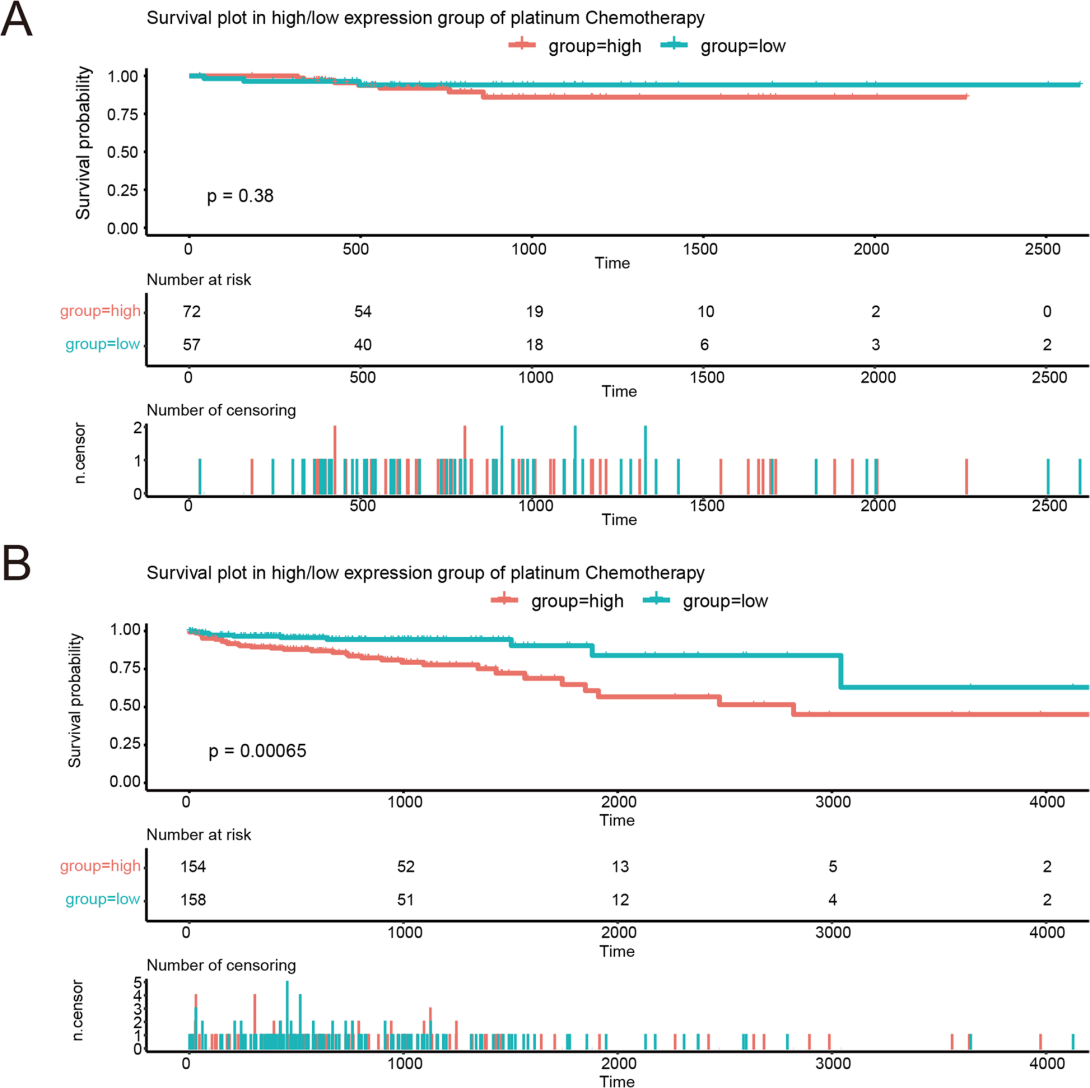
Pyroptosis risk score in response to platinum chemotherapy in CRC. **A** Overall survival plots for CRC patients with platinum-based chemotherapy in groups with 2 levels of hazard score. **B** Overall survival plots for CRC patients without platinum-based chemotherapy in groups with 2 levels of hazard score

Platinum-based chemotherapy plays an important role in CRC therapy. We asked whether pyroptosis factors are involved in the response of CRC patients to chemotherapy with platinum agents. We screened TCGA clinical data to identify sick persons with/without platinum-containing chemotherapy. Among these patients, we calculated the pyroptosis risk score. First, in the 129 patients with platinum-based chemotherapy, sick persons with a low pyroptosis points showed a trend of a higher survival rate than sick persons with a high pyroptosis points, all of whom were subjected to chemotherapy (*p* = 0.38; Fig. 7A). Second, in the 312 sick persons without platinum-based chemotherapy, sick persons with high pyroptosis points showed a significantly lower survival rate than did patients with low pyroptosis points, all of whom were not subjected to chemotherapy (*p* < 0.0001; Fig. 7B). The global survival rate of sick persons with platinum-containing chemotherapy was higher than that of those without platinum-based chemotherapy. This result suggested that pyroptosis risk score was not related to the response to platinum chemotherapy in CRC.

## Discussion

Pyroptosis is another type of lytic cell death that utilizes the gasdermin family (GSDMs), such as GSDMA-E, to form pores and generate bubble-like cell morphology. Pyroptosis plays a double part in the initiation and progression of tumors. Pyroptosis can stimulate bother normal cells to acquire malignant transformation mediated by inflammatory factors [11]. Pyroptosis, from which certain possible prognostic biomarker for tumors is derived, can also inhibit growth of tumor cells [12]. In CRC, several PRG-based models have been constructed to predict prognosis [13–16]. However, a new model as well as a comprehensive study are still been required, and our investigation was carried out to attain this purpose.

Multivariate linear regression analyses and stepwise regression analysis were utilized to establish a prognostic gene model according to 13 PRGs (AIM2, CASP1, CASP5, CASP6, CASP8, CASP9, ELANE, GPX4, GSDMD, NLRP7, NOD2, PJVK, and PRKACA), which might predict the overall survival of CRC patients. In a former study by Wei Song et al. [13], a PRG_score for forecasting recurrence-free survival (RFS) was established. Different prognostic PRG models were also developed by Jiawei Rao et al. [14], Zhicheng Zhuang et al. [15], and Chen Zheng et al. [16]. However, in our study, we first set up a new pyroptosis-associated prognostic gene model for CRC, which gives more selection for prognostic predictive in CRC.

In our research, NLRP7, which is involved in our prognostic gene model, had the highest mutation frequencies. In a previous investigation, increased protein levels of NLRP7 contributed to CRC progression and triggered polarization of M2-like macrophages [17]. This finding was consistent with our discoveries regarding NLRP7. NLRP7 is involved in inflammasome activation [18]. However, few studies have investigated the function of NLRP7 in pyroptosis. NLRP7 is overexpressed in gestational choriocarcinoma (CC) trophoblast cells and may function in an inflammasome-dependent or independent pathways [19]. In our observation, we found that NLRP7 was one of the pyroptosis-associated prognostic biomarkers in CRC. Further in vivo and in vitro investigations should be performed to clarify whether NLRP7 is implicated in pyroptosis in CRC.

An additional critical conclusion that was drawn in our study is that prognostic PRGs were remarkably correlated with the status of immune infiltration and inflammation in CRC. First, we found that the TME features and the relative fraction of immune cell infiltration differed remarkably between groups with 2 hazard scores. This result indicates an important function of PRGs in CRC tumorigenesis. A high fraction of infiltrating of B cells and plasma cells has a positive prognostic influence on CRC [20]. The low-hazard group with a better prognosis showed a higher fraction of plasma cell infiltrations, thereby suggesting a positive function of plasma cells in CRC development. It is true that antitumor responses can be created and managed in tertiary lymphoid structures (TLS) where B cells can be developed into plasma cells [21]. The infiltration of natural killer cells (NK cells) can inhibit CRC progression [22]. Moreover, infiltration of neutrophils is a beneficial prognostic factor in the early phases of CRC [23]. In our study, we discovered that the abundances of resting NK cells and neutrophils were higher in the low-hazard group than in the high-hazard group. This finding indicates that PRGs may modulate the infiltration of both cell types to affect CRC prognosis. Second, ESTIMATE scores included the immune score and stromal score. All supported the notion that immune infiltration was enriched in the low-hazard group. The immune infiltration status detected by using the ssGSEA algorithm also supported this concept. This result was consisted with reports from Song [13]. Third, PRGs (GSDME, NLRC4, etc.) were significantly associated with genes of immune infiltration. Other studies showed evidences. High levels of mononuclear cell infiltration and inflammation were found in a CRC mouse model, which shows normal GSDME expression when compared to the GSDME knockout CRC mouse model [24]. The obesity-triggered NLRC4 inflammasome was found to be activated in tumor-infiltrating myeloid cells [25]. On the other hand, PRGs (NLRP1, NLRC4, etc.) were significantly associated with inflammation genes, such as IL1R1. IL-1R1 is the receptor of interleukin-1β (IL-1β). IL-1R1(−/−) mice showed reduced NLRC4 inflammasome-dependent inflammation and IL-1β production in the lungs postinfection [26]. In addition, the expression of NLRC3, a checkpoint of inflammation, and the inflammasome components NLRP1, NLRP3, NLRC4, and AIM2 were decreased in CRC [27]. In summary, PRGs interfere with the process of immune infiltration and inflammation, thereby affecting the progression of CRC.

Our investigation has certain pitfalls. All results were produced by using the TCGA CRC cohort, and it should be nice to confirm all results by using the GEO datasets. In addition, in vitro and in vivo experimental data would be better for verifying the results.

In conclusion, we established a prognostic gene model based on 13 PRGs for CRC patients, and a comprehensive bioinformatics analysis was performed. We also found that our PRGs were significantly related to the status of immune infiltration and inflammation in CRC. Our next step would be an experimental validation investigation.

## Supplementary Information

**Additional file 1: Supplementary Table 1.** Leukocyte signature matrix (LM22).

## Data Availability

All datasets for this study are included in the TCGA (https://tcga-data.nci.nih.gov/tcga/).

## References

[1] Sung H, Ferlay J, Siegel RL (2021). Global Cancer Statistics 2020: GLOBOCAN estimates of incidence and mortality worldwide for 36 cancers in 185 countries. CA Cancer J Clin.

[2] Dariya B, Aliya S, Merchant N (2020). Colorectal cancer biology, diagnosis, and therapeutic approaches. Crit Rev Oncog.

[3] Wang YY, Liu XL, Zhao R (2019). Induction of pyroptosis and its implications in cancer management. Front Oncol.

[4] Privitera G, Rana N, Scaldaferri F (2021). Novel insights into the interactions between the gut microbiome, inflammasomes, and gasdermins during colorectal cancer. Front Cell Infect Microbiol.

[5] Wu LS, Liu Y, Wang XW (2020). LPS Enhances the chemosensitivity of oxaliplatin in HT29 cells via GSDMD-mediated pyroptosis. Cancer Manag Res.

[6] Miguchi M, Hinoi T, Shimomura M (2016). Gasdermin C is upregulated by inactivation of transforming growth factor beta receptor type II in the presence of mutated Apc, promoting colorectal cancer proliferation. PLoS One.

[7] Yu J, Li S, Qi J (2019). Cleavage of GSDME by caspase-3 determines lobaplatin-induced pyroptosis in colon cancer cells. Cell Death Dis.

[8] Erkes DA, Cai W, Sanchez IM (2020). Mutant BRAF and MEK inhibitors regulate the tumor immune microenvironment via pyroptosis. Cancer Discov.

[9] Mei Y, Xiao W, Hu H (2021). Single-cell analyses reveal suppressive tumor microenvironment of human colorectal cancer. Clin Transl Med.

[10] Ge P, Wang W, Li L (2019). Profiles of immune cell infiltration and immune-related genes in the tumor microenvironment of colorectal cancer. Biomed Pharmacother.

[11] Karki R, Kanneganti TD (2019). Diverging inflammasome signals in tumorigenesis and potential targeting. Nat Rev Cancer.

[12] Ruan J, Wang S, Wang J (2020). Mechanism and regulation of pyroptosis-mediated in cancer cell death. Chem Biol Interact.

[13] Song W, Ren J, Xiang R (2021). Identification of pyroptosis-related subtypes, the development of a prognosis model, and characterization of tumor microenvironment infiltration in colorectal cancer. Oncoimmunology.

[14] Rao J, Li W, Chen C (2021). Pyroptosis-mediated molecular subtypes and tumor microenvironment infiltration characterization in colon cancer. Front Cell Dev Biol.

[15] Zhuang Z, Cai H, Lin H (2021). Development and validation of a robust pyroptosis-related signature for predicting prognosis and immune status in patients with colon cancer. J Oncol.

[16] Zheng C, Tan Z (2021). A novel identified pyroptosis-related prognostic signature of colorectal cancer. Math Biosci Eng.

[17] Li B, Qi ZP, He DL (2021). NLRP7 deubiquitination by USP10 promotes tumor progression and tumor-associated macrophage polarization in colorectal cancer. J Exp Clin Cancer Res.

[18] Carriere J, Dorfleutner A, Stehlik C (2021). NLRP7: from inflammasome regulation to human disease. Immunology.

[19] Abi Nahed R, Elkhoury Mikhael M, Reynaud D (2022). Role of NLRP7 in normal and malignant trophoblast cells. Biomedicines.

[20] Berntsson J, Nodin B, Eberhard J (2016). Prognostic impact of tumour-infiltrating B cells and plasma cells in colorectal cancer. Int J Cancer.

[21] Sautes-Fridman C, Verneau J, Sun CM (2020). Tertiary lymphoid structures and B cells: Clinical impact and therapeutic modulation in cancer. Semin Immunol.

[22] Pan P, Kang S, Wang Y (2017). Black raspberries enhance natural killer cell infiltration into the colon and suppress the progression of colorectal cancer. Front Immunol.

[23] Wikberg ML, Ling A, Li X (2017). Neutrophil infiltration is a favorable prognostic factor in early stages of colon cancer. Hum Pathol.

[24] Croes L, Fransen E, Hylebos M (2019). Determination of the potential tumor-suppressive effects of Gsdme in a chemically induced and in a genetically modified intestinal cancer mouse model. Cancers (Basel).

[25] Kolb R, Phan L, Borcherding N (2016). Obesity-associated NLRC4 inflammasome activation drives breast cancer progression. Nat Commun.

[26] Cai S, Batra S, Wakamatsu N (2012). NLRC4 inflammasome-mediated production of IL-1beta modulates mucosal immunity in the lung against gram-negative bacterial infection. J Immunol.

[27] Liu R, Truax AD, Chen L (2015). Expression profile of innate immune receptors, NLRs and AIM2, in human colorectal cancer: correlation with cancer stages and inflammasome components. Oncotarget.

